# Metformin Ameliorates Chronic Colitis-Related Intestinal Fibrosis *via* Inhibiting TGF-β1/Smad3 Signaling

**DOI:** 10.3389/fphar.2022.887497

**Published:** 2022-05-13

**Authors:** Ying Wang, Zhi Wang, Huiping Yang, Shuze Chen, Dekai Zheng, Xiuying Liu, Qinrui Jiang, Ye Chen

**Affiliations:** ^1^ Department of Gastroenterology, State Key Laboratory of Organ Failure Research, Guangdong Provincial Key Laboratory of Gastroenterology, Nanfang Hospital, Southern Medical University, Guangzhou, China; ^2^ Department of Gastroenterology, Shenzhen Hospital, Southern Medical University, Shenzhen, China

**Keywords:** metformin, inflammatory bowel disease, intestinal fibrosis, TGF-β1/Smad3, fibroblast

## Abstract

Intestinal fibrosis is considered to be a chronic complication of inflammatory bowel disease (IBD) and seriously threatening human health. Effective medical therapies or preventive measures are desirable but currently unavailable. Metformin has been proved to have a satisfactory anti-inflammatory effects in ulcerative colitis (UC) patients. Whether metformin can ameliorate chronic colitis-related intestinal fibrosis and the possible mechanisms remain unclear. Here, we established colitis-related intestinal fibrosis in mice by repetitive administration of TNBS or DSS. Preventive and therapeutic administration of metformin to chronic TNBS or DSS colitis mice indicated that metformin significantly attenuated intestinal fibrosis by suppressing Smad3 phosphorylation. *In vitro* studies with human colon fibroblast cell line (CCD-18Co) and primary human intestinal fibroblast treated with TGF-β1 confirmed the anti-fibrotic function of metformin for fibroblast activation, proliferation and collagen production. Mechanistically, metformin particularly inhibited phosphorylation and nuclear translocation of Smad3 by blocking the interaction of Smad3 with TβRI. These findings suggest that metformin will be an attractive anti-fibrotic drug for intestinal fibrosis in future therapies.

## Introduction

Inflammatory bowel disease (IBD) is a chronic inflammatory disorder comprised of ulcerative colitis (UC) and Crohn’s disease (CD) (Maloy and Powrie, 2011). Since its precise aetiology remains unclear, it is hard to fully control the development of IBD in clinic, causing IBD patients usually progress to various severe complications such as intestinal fibrosis.

Fibrostenosis is a common complication in CD and chronic progressive UC, but its prevalence is apparently higher in CD (20% within 20 years of diagnosis) than that in UC (2–11.2%) (Rieder et al., 2017). In population-based cohorts, CD has a stricturing phenotype at initial diagnosis in 10% patients and at least 15% patients develop strictures by 10 years (Bettenworth et al., 2019). In paediatric CD patients, up to 20% of patients are discovered to have stricturing diseases at diagnosis, and increase to 40% within 10 years (Vernier-Massouille et al., 2008). These numbers, however, is likely under-estimated for most of these reports used the Montreal classification or Vienna systems that based only on patients’ symptoms but approximately 20% patients suffering small bowel stricturing CD phenotype are asymptomatic (Hansel et al., 2018). Although escalated anti-inflammatory treatments including corticosteroids, immunomodulator and anti-tumor necrosis factor (TNF) therapy have been developed to induce remission and temporary amelioration of obstructive symptoms, still 40% patients will have surgery or dilation therapy within 12 months (Bouhnik et al., 2018). Surgery, strictureplasty and endoscopic balloon dilation are the currently available therapies for CD-associated strictures and obstructions, while with 70–90% recurrence within 1 year unfortunately (Li et al., 2016). Meanwhile, surgical approaches lead to significant medical expenditure and a spiraling decline of life quality. Thus, alternative strategies targeting intestinal fibrosis have obtained substantial interests.

The pathogenesis of fibrosis in IBD develops from chronic and severe accumulation of inflammatory damage. This ability to restore integrity of tissues can result in stiffer bowel wall, intestinal stenosis and ultimately obstruction (Bessissow et al., 2018). Fibrosis development, although still not fully understood, involves the excessive deposition of collagen-rich extracellular matrix (ECM) proteins secreted by activated and numerical increased myofibroblasts at various sites of the intestinal bowel being damaged by chronically uncontrolled and exaggerated relapsing and remitting transmural inflammation (Fiocchi and Lund, 2011). Transforming growth factor β1 (TGF-β1) is a vital mediator of intestinal fibrogenesis that activates fibroblasts and stimulates anabolism of activated myofibroblasts. Agents that inhibit transforming growth factors, such as Fresolimumab, Pirfenidone or Avotermin, have been conducted in randomized controlled trials (RCTs) of patients with kidney, lung or skin fibrosis (Ferguson et al., 2009; Noble et al., 2011; Trachtman et al., 2011). TGF-β1 signal regulates gene expression through receptor-mediated activation of its canonical downstream effectors, the Smad transcription factors. Activation of the TGF-β1/Smad cascade plays a causal role in controlling gene expression of ECM proteins (Massagué, 2012).

Metformin is an oral biguanide compound widely recommended to treat type 2 diabetes mellitus (T2DM). Previous researches have proved that metformin has an extensive range of pharmacological activities apart from its antihyperglycaemic effects. Metformin was previously confirmed to attenuate bleomycin-induced pulmonary fibrosis in mice by NOX4 suppression (Sato et al., 2016). A more recent study has demonstrated that metformin resolute bleomycin-induced lung fibrosis via activating the AMP-activated protein kinase (AMPK) (Rangarajan et al., 2018). Meanwhile, hepatic protective properties of metformin have been favorably confirmed to reduce fibrosis (Fan et al., 2017). Metformin is believed to be absorbed in the intestinal walls and then concentrated in liver, inhibiting the liver gluconeogenesis to reduce blood glucose. However, a prior pharmacokinetic trial also showed that metformin was mainly concentrated in the intestine even after absorption (Wilcock and Bailey, 1994). And more recent studies have demonstrated renewed interests in the intestine as a major target organ of metformin (Rena et al., 2017). Thus, metformin might have an immense potential to directly affect intestinal fibrosis, and the underlying mechanisms also need to be studied.

In the current study, we investigated the inhibitory effects of metformin on TNBS- or DSS-treated mice models and activated intestinal fibroblasts. The inhibitory efficacies of metformin on fibroblasts including activation, proliferation and migration were evaluated. Underlying mechanisms were focused on TGF-β1/Smad3 and AMPK signaling pathways. Together, our study highlights the potential of metformin in treating patients with intestinal fibrosis.

## Materials and Methods

### Human-Derived Intestinal Specimens

Fresh human intestinal tissues were obtained from postoperative patients in Nanfang Hospital, Southern Medical University of China. Specimens were paraformaldehyde fixed and followed by paraffin embedded before finally being cut into 4 µm slices. The study design was approved by local ethical committee of Southern Medical University and informed consents were Obtained.

### Reagents and Antibodies

Metformin was obtained from Sigma-Aldrich (St Louis, MO). Recombinant human TGF-β1 (rhTGF-β1) was obtained from R&D systems (Minneapolis, MN). Aminoimidazole carboxamide ribonucleotide (AICAR, an AMPK activator) and Compound C (an AMPK inhibitor) were purchased from MCE (Monmouth Junction, NJ, United States). Primary antibodies against TGF-β1 (ab215715), TβRI (ab235578), α-SMA (ab92552), ki67 (ab16667), Smad3 (ab84177), pSmad3 (ab52903) were purchased from Abcam (Cambridge, CA, United States). Primary antibodies to Col1a1 (#72026), Vimentin (#5741), PCNA (#13110), phospho-Thr172 AMPK (#2535), AMPK (#5831) were purchased from Cell Signaling Technology (Boston, MA, United States). Anti-GAPDH and anti-Lamin A antibodies were from Proteintech (Wuhan, China). Anti-mouse and anti-rabbit IgG with conjugated horseradish peroxidase (HRP) were purchased from Fudebio (Hangzhou, China).

### Animal Experiments and Drug Treatment

Chronic inflammation and fibrosis were induced by consecutive six cycles of 0.75–2.5% 2,4,6-trinitro benzene sulfonic acid (TNBS) (Sigma-Aldrich, St Louis, MO) intrarectally initial skin presensitization with 1% TNBS in adult female BALB/c mice (appropriately 20 g) anesthetized with sodium pentobarbitalafter (Wirtz et al., 2017). Chronic dextran sulfate sodium salt (DSS) colitis was induced with 1.5% DSS (MP Biomedicals, United States) in drinking water for 7 days followed by 2 weeks of water in adult male C57BL/6 mice (appropriately 20 g). And three cycles were conducted (Scheibe et al., 2019). The animals were kept in specific pathogen-free (SPF) room with a 12-h light/dark cycle and free access to water and food for 2 weeks prior to the beginning of experiments. Mice in metformin group were treated with metformin (200 mg/kg/d) in normal saline by gavage. For preventive purposes, metformin (200 mg in 100 μl normal saline) was applied once a day by gavage during the chronic TNBS or DSS model. For therapeutic purposes, after two cycles of DSS, animals were administered intragastrically with normal saline or metformin (200 mg in 100 μl normal saline, once/day). And after the fourth cycle of chronic TNBS, metformin (200 mg in 100 μl) or normal saline was applied intragastrically once/day. All mice were euthanized at the end of experiment. A portion of colon tissues were formalin fixed and then paraffin embedded. Other colon tissues were kept at −80°C. The animal study was reviewed and approved by The Institutional Animal Care and Use Committee of Southern Medical University.

### Histopathologic Evaluation

For histopathologic analysis, colon cross-sections were stained with Hematoxylin and eosin (H&E) or Sirius Red (Sigma-Aldrich, St Louis, MO). Histologic alterations were measured with human and murine H&E sections. Collagen deposition was assessed with Sirius Red staining (Wirtz et al., 2017). The thickness of human intestinal tissue layers was evaluated by Aperio ImageScope (Aperio Technologies, Inc., United States).

### Measurement of Hydroxyproline Content

For hydroxyproline content measurement, intestine tissues were weighed before 1 ml alkaline hydrolysate was added. Samples were then hydrolyzed at 95°C for 20 min. After pH value adjustment and activated charcoal addition, samples were centrifuged at 3,500 rpm for 10 min. The supernatants obtained were then transferred to new tubes for activity measurement and hydroxyproline content was determined spectrophotometrically at 550 nm absorbance as defined in the manufacturer’s instructions (NanJing JianCheng Bioengineering Institute, China). The results were expressed as μg/mg wet weight.

### Isolation and Culture of Primary Human Intestinal Fibroblasts

We isolated primary human intestinal fibroblast cells as previously described (Johnson et al., 2016). Briefly, intestinal samples from stenosis and non-stenosis of CD patients were sterilized in Povidone Iodine Solution. Once any fat was removed, tissues were minced and incubated in sterile 0.5 mg/ml collagenase I, II and IV and 10 mg/ml DNAse at 37°C for totally 75 min under continuous rotation at 140 rpm. After incubation, the cell suspension was filtered through a 70-μm sterile mesh and centrifuged at 250 g for 10 min. The resulting supernatant was discarded, and the cell pellet was resuspended in six well, at 4 × 10^6^ cells/well with 3 ml of the Minimum Essential Medium (MEM) media, which supplemented with 1-mM sodium pyruvate, 2-mM L-glutamine and 10% heat-inactivated fetal bovine serum and 2% antibiotic-antimycotic. Cells were then grown in an atmosphere of 5% CO_2_ at 37°C.

### Cell Culture and Treatments

Human colon fibroblast cell line (CCD-18Co) was purchased from American Type Culture Collection (ATCC, Manassas, VA, United States). CCD-18Co cells were cultured in Eagle’s Minimum Essential Medium (EMEM, ATCC) medium supplemented with 10% fetal bovine serum (FBS, ExCell BIO, Shanghai, China) at 37°C, 90% humidity and 5% CO2 incubator. After starving (0% serum) for 24 h, the cells were then treated with 0.1 or 0.5 mM metformin. 4 h later, rhTGF-β1 (2 ng/ml) was added. Untreated cells and cells treated with rhTGF-β1 alone were regarded as controls.

### Immunofluorescence

Following deparaffinization and hydration, slides (human and mouse) underwent antigen retrieval by steaming in Tris-EDTA (pH 9.0) or citrate buffer (pH 6.0) for 10 min followed by permeabilizing with 0.5% Triton X-100 in PBS. Sections were then blocked with 5% bovine serum albumin (BSA, Fudebio, Hangzhou, China) in PBS for 60 min at room temperature (RT). Anti-α-SMA or anti-Vimentin antibodies were incubated overnight at 4°C and secondary goat anti-mouse or anti-rabbit antibodies were used for 1 h. Nuclei were mounted with DAPI. Images were taken from ×20 and ×40 objective fluorescence microscope.

CCD-18Co cells were fixed in 4% paraformaldehyde for 20 min and permeabilized in PBS containing 0.25% Triton X-100. Fixed cells were blocked with 5% BSA in PBS for 1 h at RT, and then stained with anti-α-SMA, anti-Vimentin, anti-Smad3 and anti-pSmad3 antibodies overnight at 4°C and with secondary antibodies for 1 h at RT. Positive stained cells were examined from ×20 objective fluorescence microscope.

### Immunohistochemical Analyses

Immunohistochemistry (IHC) was performed consistent with the manufacturer’s protocol of the universal two-step detection kit (Zhongshan Golden Bridge, Beijing, China). Slices were mounted with antibodies against TGF-β1, Col1a1, ki67 and pSmad3, and the blots were conducted with the DAB kit (Zhongshan Golden Bridge, Beijing, China). Images were taken from ×20 and ×40 objective phase contrast microscope. All control staining using PBS was provided in Supplementary Figure S1.

### Proliferation Assay

Proliferation was evaluated by 5-ethynyl-2-deoxyuridine (EdU) incorporation. After 48 h of incubation with rhTGF-β1 or metformin, cells were reincubated with EdU at 50 μM for an additional 2 h. Incorporated EdU was measured with an EdU cell proliferation detection Kit (RIBOBIO, Guangzhou, China).

### Annexin V/Propidium Iodide Apoptosis Assay

Cells were harvested and centrifuged at 1,000 g for 5 min at room temperature and the supernatant was removed. Samples were then washed with PBS and resuspended in 200 μl Annexin-V-FITC and 5 μl propidium iodide (PI) according to the manufacturer’s instructions (Annexin V-FITC staining Kit, Beyotime). The cells were protected from light and incubated for 20 min at RT. Samples were then analyzed immediately with the flow cytometer FACS ArialIII (Becton, Dickinson and Company, America). Depending on fluorescence intensity of Annexin V-FITC and PI, the cells can be distinguished into Annexin-V positive (early apoptotic cells), double positive (late apoptotic or necroptotic) cells and double negative (healthy) cells.

### Migration Assay

Transwell (8 μm pore size, Corning, NY, United States) tests were conducted for CCD-18Co migration assays. Serum-starved fibroblasts were harvested and seeded into the upper chambers with serum-free medium supplemented with rhTGF-β1 or different concentrations of metformin. The lower chambers contained EMEM with 20% FBS. Twenty-four hours later, cells that migrated to the lower surface were fixed and dyed with crystal violet solution.

### siRNA Transfection

Human-specific Smad3 siRNAs (5′-CCGCAUGAGCUUCGUCAAAdTdT-3′ and 5′-UUUGACGAAGCUCAUGCGGdTdT-3′) (Wang et al., 2018) were synthesized for knockdown of Smad3. When cells reached 60–80% confluence, they were transfected using Advanced DNA RNA Transfection Reagent (ZETA LIFE, United States) according to manufacturer’s protocol. After 24 h incubation, the culture medium was replaced by fresh EMEM media supplemented with 10% FBS for an additional 48 h. Then the cells were serum-free for 24 h, and supplemented with rhTGF-β1 (2 ng/ml) or metformin (0.5 mM) for another 48 h to be finally harvested for protein extraction.

### Isolated Extraction of Cytoplasmic and Nuclear Protein

Cytoplasmic and nuclear protein fractions were isolated by a commercially available extration kit (Beyotime, Shanghai, China) according to the manufacturer’s instructions. Protein concentrations were quantified using the bicinchoninic acid (BCA, Fudebio, Hangzhou, China) protein assay.

### Protein Extraction and Western Blot

Cell lysates or intestine homogenates was prepared with RIPA buffer, protease and phosphatase inhibitors (Fudebio, Hangzhou, China). After SDS-PAGE separation, PVDF membranes were incubated with specific antibodies as described above followed by probed with HRP-conjugated goat anti-mouse or anti-rabbit IgG.

### Co-Immunoprecipitation

The interaction within TβRI and Smad3 was determined by co-immunoprecipitation (CO-IP). Cells were lysed and harvested in CO-IP buffer supplemented with protease inhibitor and centrifuged at 14,000 g for 15 min at 4°C. Soluble cell lysates were then incubated with anti-Smad3 antibody at 4°C overnight with constant agitation, and followed by Protein A or G beads (Proteintech, Wuhan, China) incubation at 4°C for 4 h. The precipitated complexes were then washed with CO-IP buffer three times and boiled for 5 min and finally subjected to Western blot analysis.

### Statistical Analysis

Statistical analysis was carried out on GraphPad Prism 7 (GraphPad Software, CA, United States). Student’s t-test was utilized for comparisons of two groups, and one-way ANOVA was used for multigroup comparisons. Data are demonstrated as means ± standard deviation (SD). *p* < 0.05 was considered as statistical significance.

## Results

### Preventive Metformin Treatment Attenuates Chronic Colitis-Related Intestinal Fibrosis Induced by 2,4,6-Trinitro Benzene Sulfonic Acid

**FIGURE 1:**
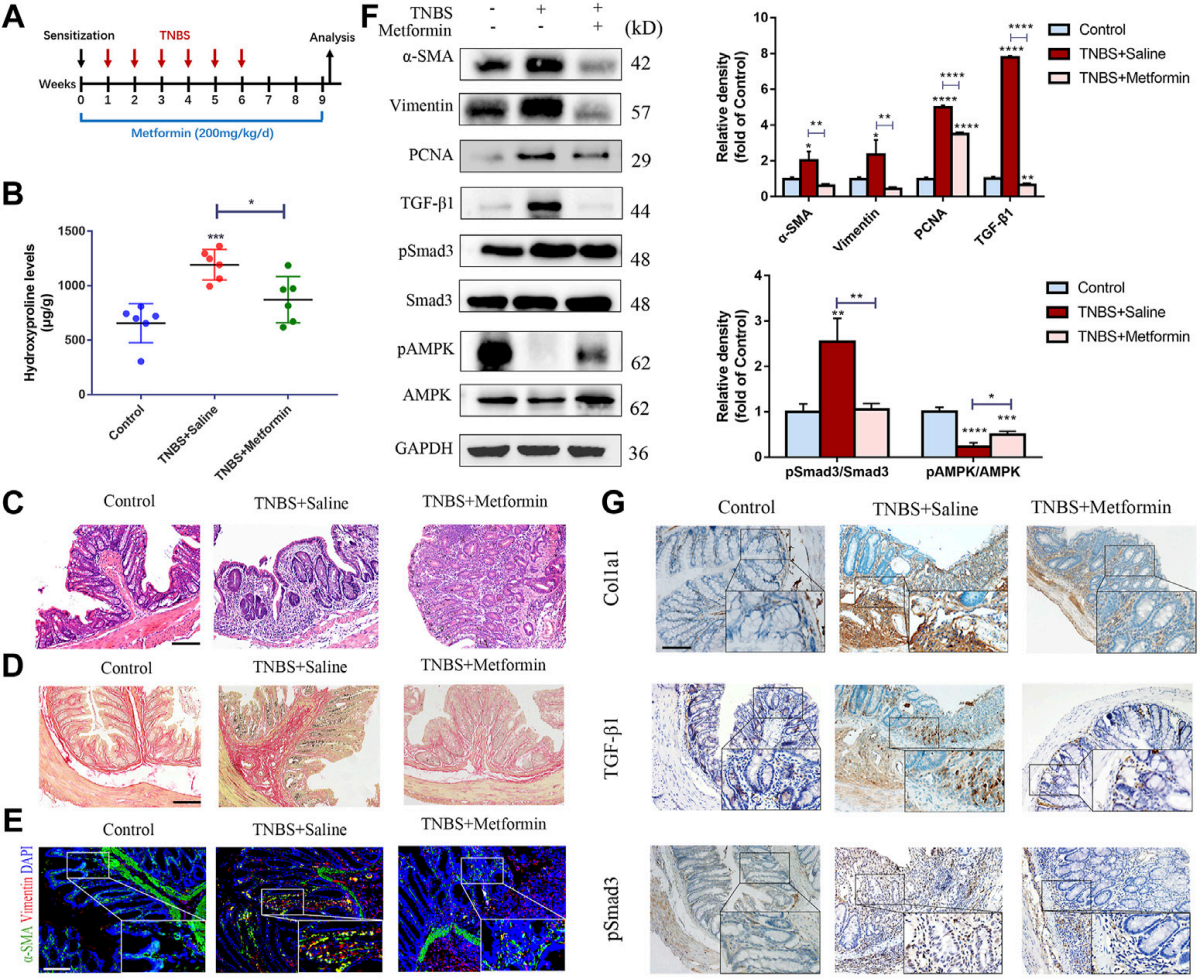
Preventive metformin application decreases intestinal fibrosis in TNBS mice model. **(A)** Mice experimental protocol of TNBS colitis. **(B–D)** Colon tissues were studied by **(B)** Hydroxyproline assay, **(C)** H&E staining or **(D)** Sirius Red staining at week nine *ex vivo* (*n* = 6/group). **(E)** IF for α-SMA and Vimentin were conducted in colon sections of three groups (*n* = 6/group). **(F)** Representative images of western blot showing changes of the key fibrotic markers and proteins in TGF-β1/Smad3 pathway after metformin treatment. Quantitative analyses of western blot (*n* = 3 independent experiments) were shown **(G)** Representative micrographs of immunohistochemical staining for Col1a1, TGF-β1 and pSmad3 in colon specimens. Scale bars, 100 μm. Quantitative results were analyzed by one-way ANOVA test. * *p* < 0.05; ** *p* < 0.005; *** *p* < 0.0005.

To detect the potential preventive effects of metformin, we daily applied metformin to TNBS model of intestinal fibrosis by gavage (Figure 1A). Inflammatory changes established by H&E staining of colon slices from the TNBS group indicated massive inflammatory cells and distorted crypt structures, while metformin treatment attenuated these histopathologic alterations (Figure 1C). We evaluated colonic content of hydroxyproline to examine the colon fibrotic levels. And we found that hydroxyproline level was markedly higher in TNBS-treated group than in control group, while metformin treatment attenuated it significantly (Figure 1B). Furthermore, Sirius Red staining showed strong collagen fiber deposition in TNBS group, which was obviously reduced by metformin treatment (Figure 1D). During fibrosis, activated colonic fibroblasts, known as myofibroblasts can secrete abundant fibrosis makers, such as α-SMA and Col1a1, which directly contribute to ECM proliferation and fibrogenesis. To determine whether metformin can prevent the fibroblasts activation, proliferation and subsequent fibrotic proteins expression, we conducted immunofluorescence, western blot and immunohistochemical assays. Initially, we showed that activated fibroblasts that co-expressing α-SMA and Vimentin were increased in TNBS-treated mice and decreased after metformin treatment, as illustrated by IF (Figure 1E). Then we conducted western blot assays to evaluate protein expression of α-SMA, Vimentin and PCNA. Correspondingly, these proteins increased in TNBS-treated group as compared with the control, while metformin was confirmed to be able to inhibit the protein expression (Figure 1F). And immunohistochemical staining indicated an enhanced expression of Col1a1 in mice with chronic colitis induced by TNBS compared to the control ones. And metformin-treated mice indicated a significant decrease in Col1a1 expression (Figure 1G).

### Preventive Metformin Treatment Attenuates Chronic Colitis-Related Intestinal Fibrosis Induced by Dextran Sulfate Sodium Salt

**FIGURE 2:**
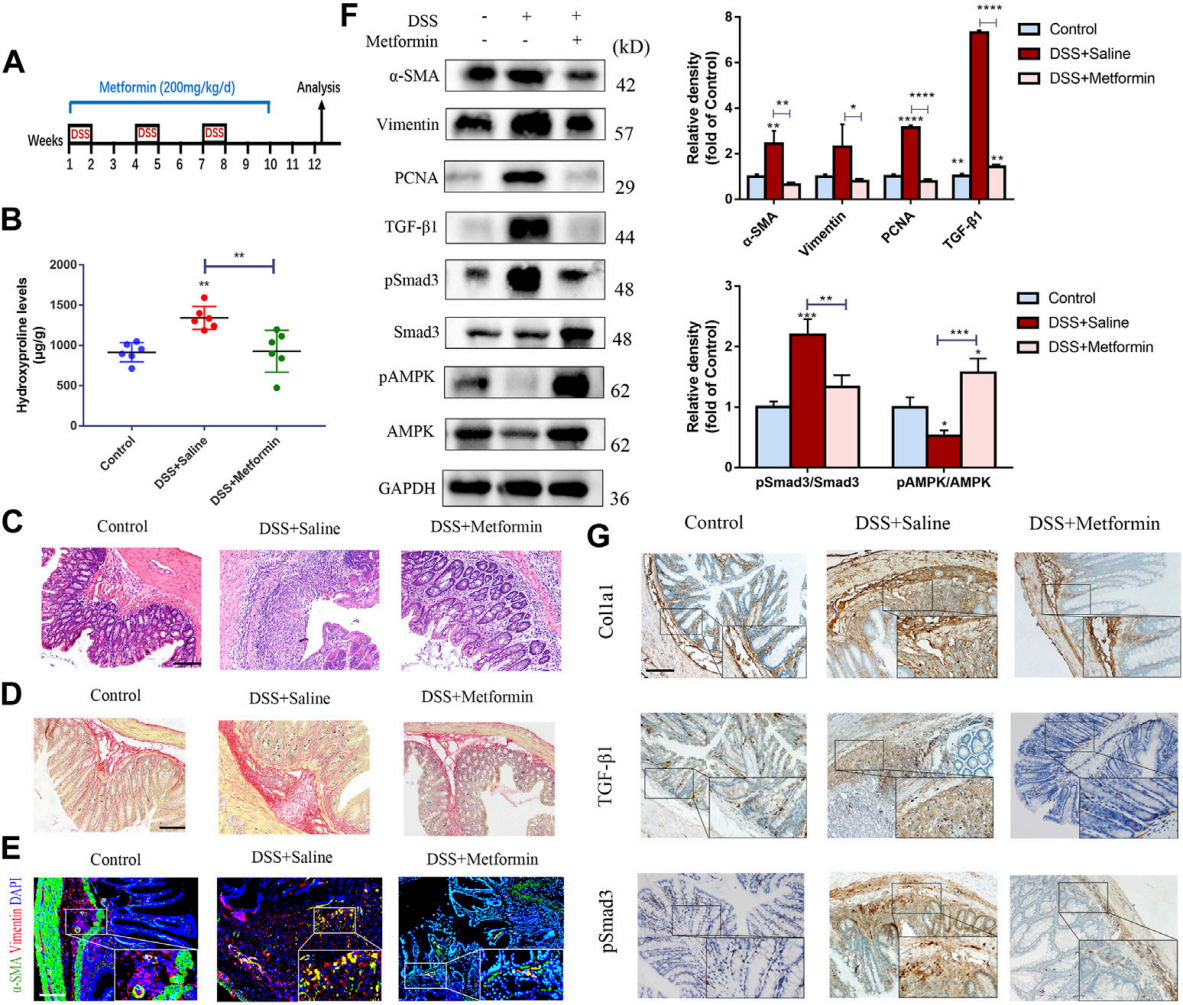
Preventive metformin application decreases intestinal fibrosis in DSS mice model. **(A)** Mice experimental protocol of DSS colitis. **(B–D)** Colon tissues were analyzed by **(B)** Hydroxyproline assay, **(C)** H&E staining or **(D)** Sirius Red staining (*n* = 6/group). **(E)** IF for α-SMA and Vimentin was analyzed in colon slices of three groups (*n* = 6/group). **(F)** Representative pictures of western blot indicating changes of the key fibrotic markers and proteins in TGF-β1/Smad3 signaling after metformin treatment. Quantitative analyses of western blot (*n* = 3 independent experiments) were shown **(G)** Representative images of immunohistochemical staining for Col1a1, TGF-β1 and pSmad3 in colon specimens. Scale bars, 100 μm. Quantitative results were analyzed by one-way ANOVA test. * *p* < 0.05; ** *p* < 0.005; *** *p* < 0.0005.

Next, we analyzed the preventive effects of metformin in the chronic DSS model (Figure 2A). We observed that metformin reduced the elevation of inflammation infiltration signs (Figure 2C) and collagen accumulation (Figures 2B,D) induced by DSS treatment. IF analysis showed reduced number of activated colonic fibroblasts in metformin-treated mice (Figure 2E). Again, expression of α-SMA, Vimentin and PCNA was significantly diminished in metformin-treated mice compared to controls treated with DSS alone (Figure 2F). Finally, Col1a1 expression was also found lowered in tissues from the metformin-treated chronic DSS model, as indicated by IHC staining (Figure 2G).

To investigate the underlying mechanisms of the antifibrogenic function of metformin, we evaluated the effects of metformin on TGF-β1/Smad3 signaling, a major profibrotic pathway. As shown in Figures 1E, 2E, western blot analysis indicated that the expression of TGF-β1 and pSmad3 was significantly elevated in TNBS- and DSS-treated mice as compared with normal controls (Figures 1F, 2F). Metformin treatment markedly inhibited pSmad3 expression in fibrosis mice, consistent with the results of IHC assay (Figures 1G, 2G). These data showed that metformin could inhibit activated TGF-β1/Smad3 pathway in colitis-related intestinal fibrosis.

It is well acknowledged that metformin can activate adenosine monophosphate-activated protein kinase (AMPK), an important metabolic regulator and cellular bioenergetic sensor. Not surprisingly, Western blot analysis indicated that preventive treatment of metformin increased the phosphorylated AMPK (pAMPK) levels in two different mice models of intestinal fibrosis (Figures 1F, 2F).

### Metformin Treatment Reverses Established Intestinal Fibrosis Induced by 2,4,6-Trinitro Benzene Sulfonic Acids and Dextran Sulfate Sodium Salt in Mice

**FIGURE 3:**
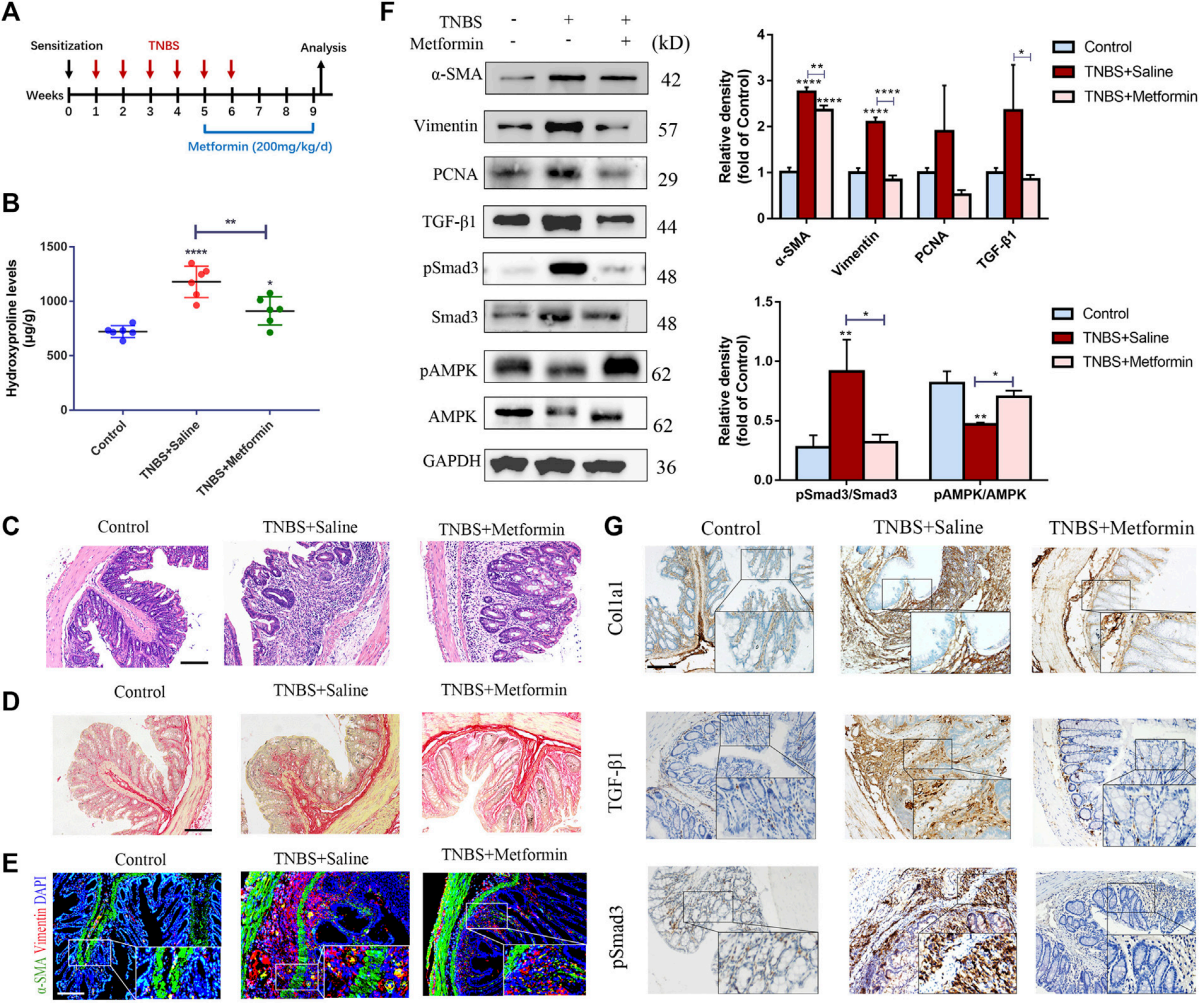
Therapeutic metformin application attenuates intestinal fibrosis in TNBS mice model. **(A)** Mice experimental protocol of TNBS colitis. **(B–D)** Colonic tissues were analyzed with **(B)** Hydroxyproline assay, **(C)** H&E staining and **(D)** Sirius Red staining at week nine (*n* = 6/group). **(E)** IF staining for α-SMA and Vimentin were conducted in colon slices (*n* = 6/group). **(F)** Expression of fibrotic and proliferative markers was analyzed via western blot of colon tissues. Relative density of each protein to GAPDH and pSmad3/Smad3 ratios are shown. **(G)** Colon tissue specimens of mice were stained by IHC for Col1a1, TGF-β1 and pSmad3. Scale bars, 100 μm. Quantitative results were analyzed by one-way ANOVA test. * *p* < 0.05; ** *p* < 0.005; **** *p* < 0.0001.

**FIGURE 4:**
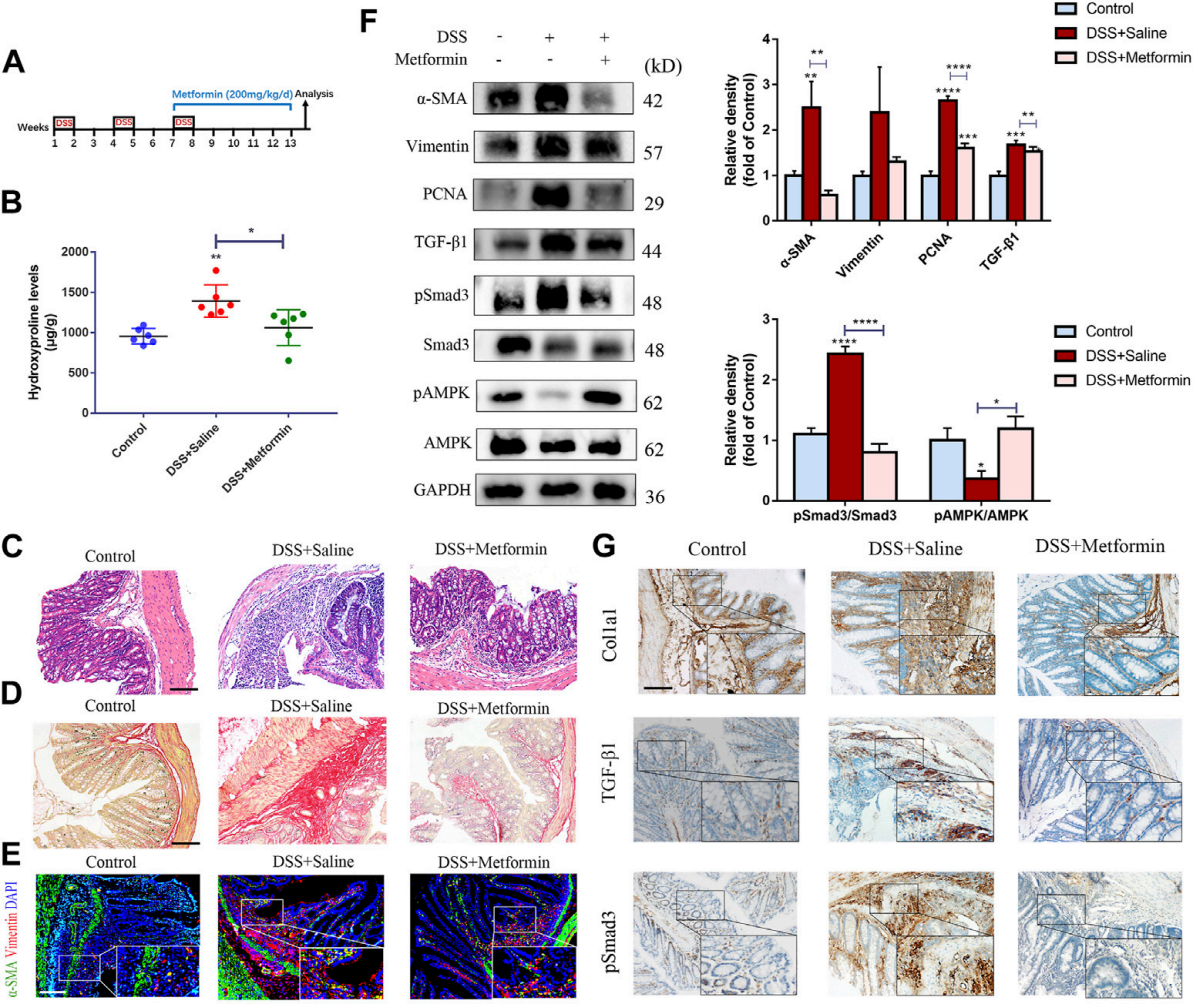
Therapeutic metformin application attenuates intestinal fibrosis in DSS mice model. **(A)** Mice experimental protocol of DSS colitis. **(B–D)** Colonic tissues were analyzed by **(B)** Hydroxyproline assay, **(C)** H&E staining and **(D)** Sirius Red staining (*n* = 6/group). **(E)** Colonic tissues of mice were stained with α-SMA and Vimentin **(F)** α-SMA, Vimentin, TGF-β1, pSmad3 and Smad3 expressions were evaluated in colonic tissue sections by western blot. And relative density of each protein to the levels of GAPDH and pSmad3/Smad3 ratios are shown. **(G)** IHC analysis was conducted (*n* = 6/group). Scale bars, 100 μm. Quantitative results were analyzed by one-way ANOVA test. * *p* < 0.05; ** *p* < 0.005; **** *p* < 0.0001.

To confirm whether metformin could affect already established intestinal fibrosis, metformin application was started on the chronic TNBS and DSS models at the time points that intestinal fibrosis had already been established (Figures 3A, 4A). In the chronic TNBS model with developed fibrosis, mice given with metformin showed attenuated mucosal inflammation infiltration and reduced intestinal fibrosis (Figures 3C,D). The hydroxyproline level was also strikingly reduced upon metformin treatment (Figure 3B). Consistent with that, the accumulation of myofibroblasts (α-SMA^+^ Vimentin^+^ cells) and the expression of fibrotic markers, proliferative proteins were potently diminished upon metformin application as compared with TNBS-treated group (Figures 3E–G).

In a second part, metformin was administrated to mice with established intestinal fibrosis in the chronic DSS model by gavage (Figure 4A). In line with the TNBS model, we also discovered reduced inflammation, decreased hydroxyproline level, diminished submucosal collagen deposition, reduced numbers of myofibroblasts and lowered expression of fibrotic and proliferative proteins (Figures 4B–G).

To further confirm the inhibitory effect of metformin on TGF-β1/Smad3 pathway in preestablished fibrosis, we evaluated the expression of these proteins again. Metformin inhibited the protein expression of TGF-β1 and pSmad3 significantly in mice that had already developed fibrosis (Figures 3F, 4F), which was paralleled with the IHC images (Figures 3G, 4G). And therapeutic treatment of metformin upregulated the pAMPK levels again (Figures 3F, 4F).

### Metformin Inhibits TGF-β1-Induced Activation, Collagen Production and Proliferation of Human Colon Fibroblasts *in Vitro*

**FIGURE 5:**
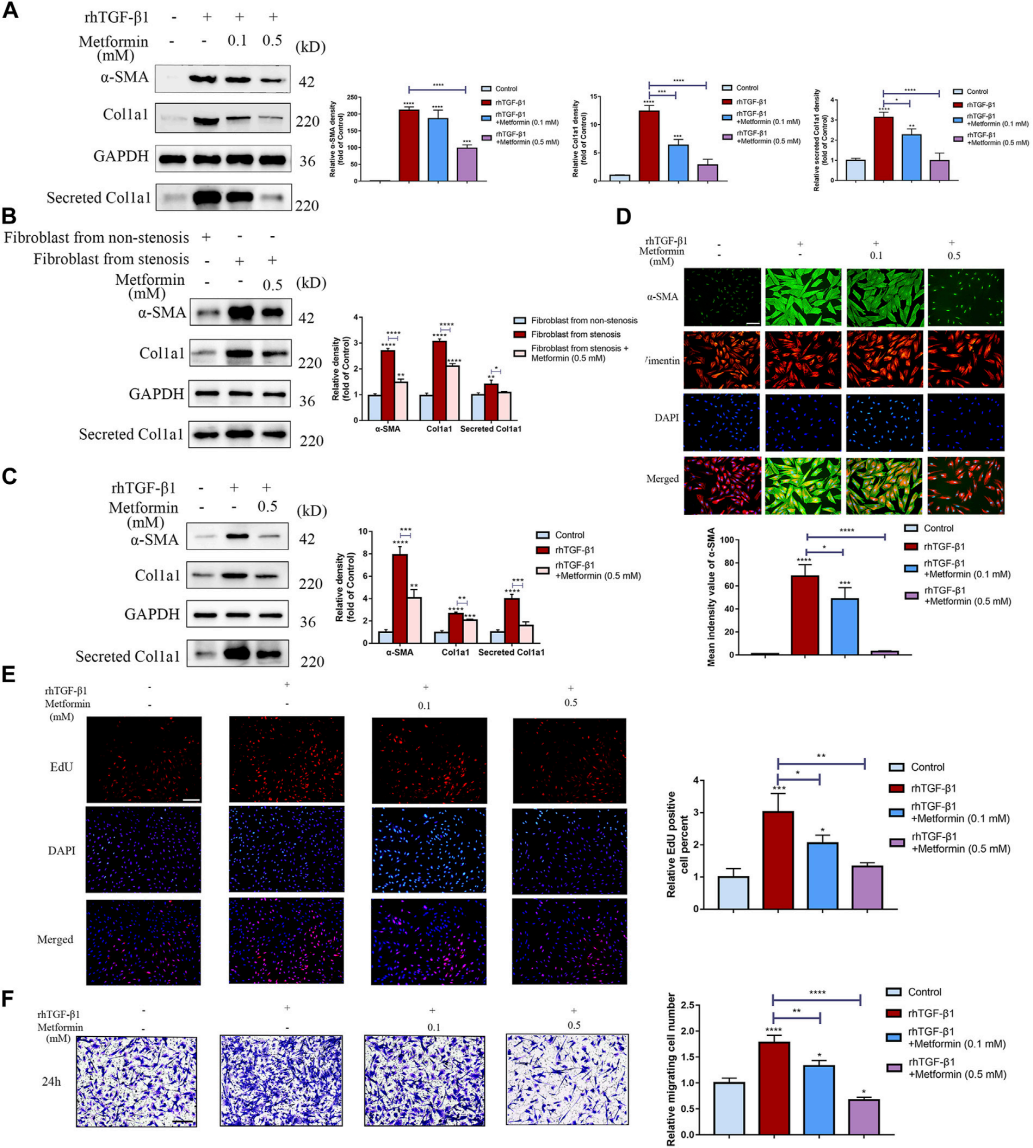
Metformin dose-dependently attenuates TGF-β1-induced collagen production and proliferation in cultured human colon fibroblasts. **(A)** Protein expressions of Col1a1 and α‐SMA in CCD-18Co cells induced by TGF-β1 or metformin, and Col1a1 in supernatant secreted by CCD-18Co cells. Relative densitometry values are indicated beside blots as ratios relative to GAPDH **(B)** Protein expressions of Col1a1, α‐SMA and Col1a1 secretion in primary intestinal fibroblasts treated by metformin. Relative densitometry values are indicated beside blots as ratios relative to GAPDH. **(C)** Protein expressions of Col1a1, α‐SMA and Col1a1 secretion induced by TGF-β1 or metformin in primary intestinal fibroblasts isolated from non-stenosis. Relative densitometry values are indicated beside blots as ratios relative to GAPDH. **(D)** CCD-18Co cells stimulated by TGF-β1 or metformin were stained with primary antibodies against Vimentin and α‐SMA. Representative immunofluorescent-stained images of Vimentin and α‐SMA expression in different groups are shown. **(E)** Proliferation of human colon fibroblasts treated with or without metformin was demonstrated by EdU incorporation in response to 2 ng/ml rhTGF-β1. Representative IF images are shown. **(F)** Cell motility was monitored by Transwell tests (*n* = 3 independent experiments). Representative microscopy pictures (left) and quantifications of migrating cells (right) are shown (* *p* < 0.05; ** *p* < 0.005; *** *p* < 0.0005; **** *p* < 0.0001). Means ± SD. Scale bars, **(D)** 100 μm; **(E,F)** 200 µm.

We initially investigated the inhibitory response of metformin on production of the extracellular matrix (ECM) genes in TGF-β1-treated CCD-18Co cells. To assess the dose-dependent bioactivity of metformin and select the optimal concentration of metformin, a dose-response experiment on CCD-18Co cells was performed at 0.1 and 0.5 mM concentrations. Pre-exposure to metformin at a relatively higher concentration (0.5 mM) markedly inhibited Col1a1 and α-SMA expression in CCD-18Co cells. Meanwhile, it inhibited the secretion of Col1a1 in supernatant (Figure 5A). But metformin at a relatively lower concentration (0.1 mM) did not alter α-SMA expression. These results show that the dosage of metformin has an essential effect on its bioactivity. To further evaluate the effect of metformin on human intestinal fibroblast, we isolated primary intestinal fibroblasts from the stenosis and non-stenosis of CD patients. Interestingly, fibroblasts from the stenosis had a higher expression of α-SMA and Col1a1, meanwhile secreted more Clo1a1 in supernatant than fibroblasts isolated from non-stenosis (Figure 5B). And metformin at 0.5 mM significantly diminished the expression of these proteins (Figure 5B). And we also found that metformin at 0.5 mM markedly decreased the up-regulation of α-SMA and Col1a1 induced by TGF-β1 of fibroblast isolated from non-stenosis of CD patients (Figure 5C).

α-SMA is a crucial marker of myofibroblasts and to further examine the role of metformin in myofibroblast differentiation, co-immunofluorescent staining was performed and revealed that α-SMA protein was significantly increased in TGF-β1-treated CCD-18Co cells and the effect was diminished after metformin treatment (Figure 5D). Another potential anti-fibrotic mechanism of metformin is attenuating proliferation of colon fibroblasts. EdU incorporation was further conducted to show that metformin had an inhibitory response on the proliferation of colon fibroblasts (Figure 5E). Meanwhile, Annexin V/Propidium Iodide apoptosis assay indicated that metformin at 0.5 mM didn’t promote apoptosis of fibroblast (Supplementary Figure S2). Moreover, metformin of lower or higher dosage both inhibited cell motility of myofibroblasts (Figure 5F), but higher dosage could be more significant.

### Metformin Blocks the Profibrotic Transforming Growth Factor-Beta 1/Small Mothers Against Decapentaplegic 3 Signaling Pathway in Cultured Human Colonic Fibroblasts

**FIGURE 6:**
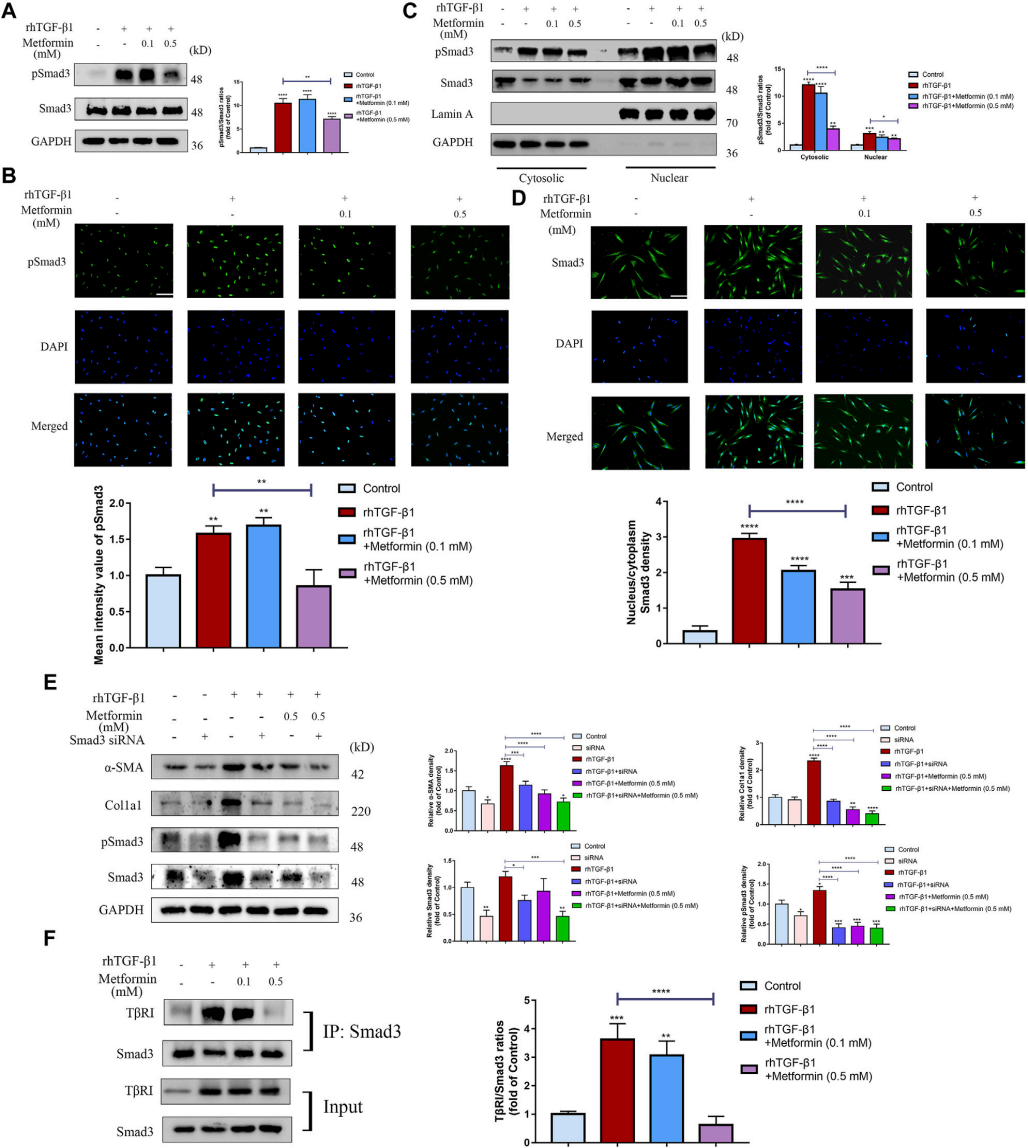
Metformin blocks TGF-β1/Smad3 signaling in cultured human colonic fibroblasts. **(A)** Representative western blot graphs of pSmad3 indicate that metformin inhibits TGF-β1-induced Smad3 phosphorylation in CCD-18Co cells. Relative densitometric ratios of pSmad3 to Smad3 are shown. **(B)** CCD-18Co cells induced by TGF-β1 were immunocytochemically stained with primary pSmad3 antibody. Representative ICC staining images are shown. And mean intensity quantitation of pSmad3 in CCD-18Co cells was performed. **(C)** Western blots analyses show that metformin suppresses the increase of pSmad3 in the cytoplasm and nuclear stimulated by TGF-β1. Subcellular nuclear and cytosolic fractions were analyzed by western blot for pSmad3 and Smad3. The blots were analyzed with antibodies against Lamin A or GAPDH to confirm identical protein loadings to further assess the relative content of the nuclear and cytosolic fractions (*n* = 3 independent experiments). Relative densitometry values of pSmad3 are shown besides the blots as ratios relative to Smad3. **(D)** Representative Immunofluorescence graphs show that metformin suppresses nuclear translocation of Smad3 stimulated by TGF-β1. Green signals represent the Smad3 protein. And blue fluorescence signals represent the nucleus. Intensity nucleus/cytoplasm ratios of Smad3 was performed. **(E)** After being infected with scrambled or Smad3-specific siRNA, TGF-β1‐induced CCD-18Co cells were further treated with metformin. Protein expression of α-SMA, Col1a1, pSmad3 and Smad3 were shown by western blot analysis. Relative density of each protein to GAPDH are shown. **(F)** CCD-18Co cells were treated with 0.1 or 0.5 mM metformin and/or 2 ng/ml TGF-β1 for 48 h. Cell lysates were IP with anti‐Smad3 antibody (*n* = 3 independent experiments) and TβRI was detected by western blot. Relative densitometry values of TβRI are shown beside the blots as ratios relative to Smad3. Scale bars, **(B,D)** 400 µm.

To further determine the underlying mechanism on inhibition of TGF-β1-induced cell activation and collagen synthesis by metformin, we examined the expression of Smad3, one of the main downstream molecules of TGF-β1 activation in fibroblasts. Western blot analysis revealed that TGF-β1 (2 ng/ml) treatment markedly increased Smad3 phosphorylation level in CCD-18Co cells. Treatment with metformin (0.5 mM) inhibited this process (Figure 6A). Meanwhile, metformin didn’t alter total Smad3 levels in colonic fibroblasts. However, Metformin of low dosage (0.1 mM) did not reduce TGF-β1-induced Smad3 phosphorylation (Figure 6A), corresponding with the results of Immunofluorescent analysis (Figure 6B). Nuclear translocation of Smads is one of the major components regulating the TGF-β1/Smads signaling. Western blot analysis indicated that metformin (0.5 mM) significantly downregulated the pSmad3 level stimulated by TGF-β1 both in the cytoplasm and nucleus (Figure 6C), which showed that the blocking effect of metformin occurs before phosphorylation of Smad3. Immunofluorescent staining was also applied to assess whether metformin inhibited nuclear translocation of Smad3. In vehicle-treated CCD-18Co cells, staining of Smad3 (Figure 6D) was distributed almost evenly in CCD-18Co cells. This demonstrated that significant Smad3 nuclear translocation did not occur. Treatment with TGF-β1 (2 ng/ml) markedly induced Smad3 nuclear translocation as shown by strong Smad3 immunostaining density in the nucleus (Figure 6D). Treatment with metformin (0.5 mM) significantly blocked TGF-β1-stimulated Smad3 translocation (Figure 6D). To further evaluate the effect of metformin in Smad3 phosphorylation, we tested the effect of Smad3 knockdown on fibroblast activation and collagen production using siRNA in TGF-β1-induced CCD-18Co cells. Western blot analysis demonstrated that Smad3 protein was markedly lower in knocked-down cells compared with that in scrambled siRNA-infected cells (Figure 6E). Furthermore, expression of α-SMA, Col1a1 and pSmad3 was decreased by siRNA-mediated Smad3 knockdown both in the presence and absence of TGF-β1 stimulation (Figure 6E). Compared with Smad3 suppression in the presence of TGF-β1 stimulation, metformin also had a significant and similar effect on α-SMA, Col1a1 and pSmad3 protein expression (Figure 6E). Meanwhile, co-operated with the Smad3 interference, metformin further down‐regulated the expression of α-SMA, Col1a1 and pSmad3 (Figure 6E). Moreover, to illustrate the mechanisms of how metformin directly affects Smad3 phosphorylation, we conducted the CO-IP experiment. And we indicated that metformin of a relatively higher dosage significantly disrupted the TGF-β1-induced interaction between Smad3 with TβRI (Figure 6F). These data further show that metformin exert anti-fibrotic effects through specific effects on the phosphorylation of Smad3. Therefore, metformin can serve as a potent inhibitor of the TGF-β1/Smad3 signaling.

### Inhibitory Effects of Metformin on Cell Activation and Collagen Synthesis Are Independent of AMP-Activated Protein Kinas Activation

**FIGURE 7:**
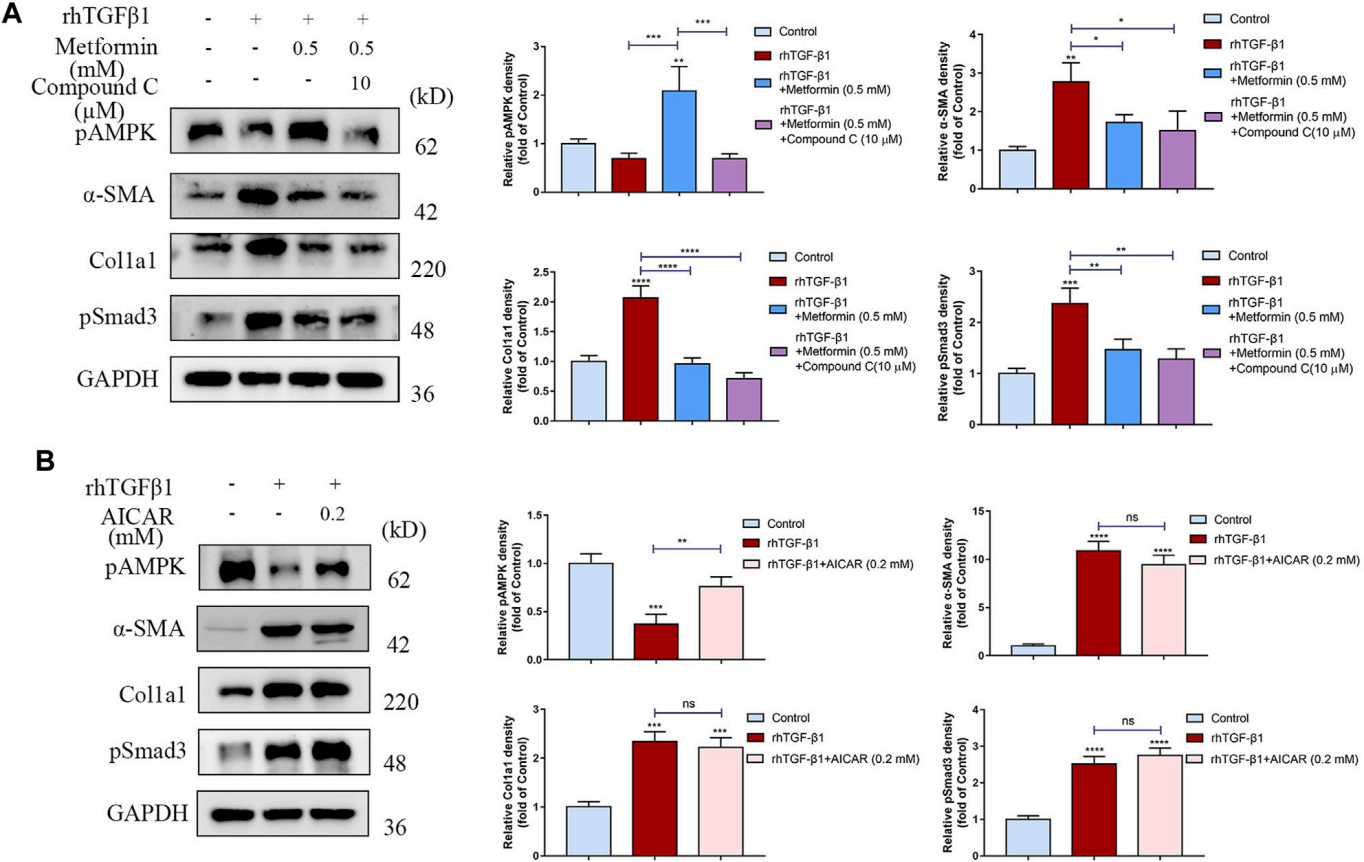
The anti-fibrotic effects of metformin are independent of AMPK. **(A)** Compound C, an AMPK inhibitor, does not reverse the anti-fibrotic effects of metformin. Relative density values are calculated beside the blots. **(B)** Another AMPK activator, AICAR (0.2 mM), does not suppress the cell activation and collagen synthesis stimulated by TGF-β1. Relative density values are shown beside the blots.

We then determined whether the anti-fibrotic effect of metformin was dependent on AMPK activation. We used Compound C, an AMPK inhibitor, and found that the inhibition of fibroblast activation and collagen synthesis by metformin wasn’t reversed (Figure 7A). Additionally, 5-aminoimidazole-4-carboxamide riboside (AICAR), another AMPK activator, did not suppress cell activation and collagen synthesis stimulated by TGF-β1 in cultured human colonic fibroblasts (Figure 7B). Together, these results indicated that metformin could inhibit intestinal fibrosis via an AMPK-independent manner.

## Discussion

Current studies have suggested improved insights into the mechanisms of intestinal fibrosis (Wang et al., 2021). However, medical therapeutic approaches for intestinal strictures still remain limited and rely primarily on multiple surgical or endoscopic interventions. Recent anti-inflammatory therapies, chiefly anti-TNF (tumor necrosis factor) agents, have not been proven to be effective in preventing intestinal fibrosis (Lin et al., 2021). And crescent evidence has shown that underlying mechanisms that regulate intestinal fibrosis appear to be separated from those that regulate inflammation (Chan et al., 2018) and this might be the reason why strictures could present several years after the remission of active inflammation. Thus, development of specific anti-fibrotic medicine is a critical unmet clinical need. In the present study, we have identified: 1) metformin could prevent and reverse intestinal fibrosis induced by TNBS and DSS; 2) the anti-fibrotic mechanisms of metformin may attribute to the suppressions on fibroblasts activation and collagen synthesis; 3) metformin blocks the TGF-β1/Smad3 signaling through inhibiting the interaction of TβRI and Smad3, finally suppresses the Smad3 phosphorylation and nuclear translocation; and 4) the anti-fibrotic functions of metformin might be independent of AMPK activation.

The mechanisms that induce excessive ECM accumulation and fibrogenesis in the intestine are considered to be comparable with those induce fibrosis in other organs. Physiologic healing mechanisms, which compensate for damaged tissues, involve a controlled inflammatory response mediated by ECM and mesenchymal cells (Latella et al., 2014). Otherwise, fibrosis is an abnormal exaggerated repairing program characterized by excessive deposition of collagen-rich ECM, produced mainly by the transient or permanent numerically expanded mesenchymal cells, including myofibroblasts, the main mediators of fibroplasia in gut (Rieder et al., 2017; Lawrance et al., 2017). TGF-β/Smads pathway is a central mediator during a variety of organ fibrogenesis, also the most pivotal inducer of activation of fibroblasts (Massagué, 2012). At the cell surface, TGF-β isoforms bind a heterotetrameric complex of transmembrane serine/threonine kinases receptors (types I and II) and then induce the transphosphorylation from the type II kinases receptor to the type I kinases receptor. Activated type I receptor phosphorylates selected R-Smads (receptor-activated Smads) at C-terminal serines, and these R-Smads (generally Smad2 and Smad3) then form a trimeric complex with common Smad4 (Co-Smad) (Derynck and Zhang, 2003). In the development of organ fibrosis, these activated Smad complexes then translocated into the nuclei, where they accumulate and regulate the transcriptions of target pro-fibrotic genes, which finally induce the major fibrotic reactions (Macias et al., 2015). In CD patients, these mechanisms and factors ultimately result in the pathologic thickness of the intestinal wall. As described in previous studies, we observed a marked thickening of submucosa and significant accumulation of collagen in stenotic tissues of CD patients (Supplementary Figures S3A–D). Moreover, fibrotic tissues in CD were characterized by an overexpression of proteins in TGF-β1/Smad3 signaling (Supplementary Figures S3E,F) and expansion of α-SMA^+^ Vimentin^+^ myofibroblasts, a main cell type known to produce collagen in fibrogenesis (Supplementary Figure S3G).

In our previous study, we proved that metformin exerted anti-inflammatory effects and protected intestinal barrier in colitis (Deng et al., 2018). Given the anti-fibrotic role of metformin in other organs, particularly in lung (Sato et al., 2016; Rangarajan et al., 2018; Kheirollahi et al., 2019), we believe that metformin might exert protective effects on intestinal fibrosis. In the present study, we indicated anti-fibrotic role of preventive metformin (200 mg/kg/day) treatment in chronic TNBS or DSS mice models. And we also found that metformin (200 mg/kg/day) reversed the established intestinal fibrosis and had a therapeutic effect. Metformin dosage that used in type 2 diabetes patients is 10–40 mg/kg (Xiao et al., 2010). Due to the difference of body surface area between human and mice, the appropriate dose of metformin used in mice is 120–480 mg/kg. Then we chose a relative lower dosage of 200 mg/kg/day. And the dosage of metformin we applied in this study was proved to be effective on intestinal fibrosis. These findings also suggested that metformin at low doses might inhibit intestinal fibrosis in humans, meanwhile minimize the side-effects and improve the clinical feasibility.

It is well accepted that myofibroblasts produce expanded amounts of ECM components during chronic tissue fibrosis. Myofibroblasts are considered the main promoters of fibrogenesis in gut (Li et al., 2019). To explore the mechanisms of the anti-fibrotic effects of metformin, we investigated the effects of metformin on activation, proliferation and mobility of human colon fibroblasts. TGF-β1 has been reported to stimulate myofibroblast differentiation and collagen production *in vivo* and *in vitro*, thereby contributing to intestinal fibrosis. In our study, metformin inhibited the increase of TNBS- or DSS-induced TGF-β1 expressions. Furthermore, we observed that metformin dose-dependently blunted TGF-β1-induced α-SMA, Col1a1 protein expression as well as the secretion of Col1a1 in CCD-18Co cells and primary human intestinal fibroblasts. Similarly, some studies also demonstrated that metformin suppressed cardiac (Bai et al., 2013), lung (Cheng et al., 2021) fibrosis by inhibiting fibroblast activation. With regard to proliferation, metformin has been suggested to inhibit the growth of many cell types, such as smooth muscle cells (Zhou et al., 2020), liver and breast cancer cells (Yan et al., 2020). We also demonstrated that metformin had inhibitory effects on the proliferation of colonic myofibroblasts. Together, the anti-fibrotic mechanisms of metformin may be mainly attributable to the suppression of TGF-β1-induced cell activation, proliferation and mobility.

As mentioned above, TGF-β/Smads pathway played a critical role in the pathogenesis of human intestinal fibrosis. In the present study, we showed that treatment with metformin dramatically inhibited the rise of pSmad3 both *in vivo* and *in vitro*. We found that metformin treatment suppressed TGF-β1-induced phosphorylation and nuclear translocation of Smad3. And to further evaluate the effects of metformin in inhibiting pSmad3, we knocked down the Smad3 expression by siRNA in TGF-β1-induced CCD-18Co cells. After depletion of Smad3 expression by RNA interference, the inhibitory effects of metformin on α-SMA, Col1a1 and pSmad3 were further aggravated. These results demonstrated that the effects of metformin on attenuating the expression of fibrotic markers were as significant as directly knocking down the Smad3 expression. And they created a synergistic effect on inhibiting intestinal fibrosis. As mentioned above, the generation of pSmad3 stimulated by the interaction of Smad3 and TβRI is a key step in TGF-β1/Smad3 pathway. It had been reported that in melanoma, metformin suppressed phosphorylation of Smad3 by binding with the TGF-β1 extracellularly and meanwhile reduced the interactions of TβRI and Smad3 intracellularly (Li et al., 2018). To further uncover the mechanisms by which metformin specifically inhibited phosphorylation of Smad3 in intestinal fibrosis, we investigated the effects of metformin on the interaction TβRI with Smad3. And the co‐immunoprecipitation analysis illuminated that metformin reduced the binding of Smad3 with TβRI. Thus, we concluded that metformin played an anti-fibrosis role by blocking the interaction between TβRI and Smad3.

Metformin is the best-known activator of AMPK in clinic. Our results demonstrated that continuous application of metformin markedly activated AMPK *in vivo*. Several studies have showed that metformin confers anti-fibrotic actions in renal (Lee et al., 2018), lung (Rangarajan et al., 2018), left ventricular (Asensio-Lopez et al., 2018) fibrosis through AMPK activation. Strikingly, at the cellular level, we found that Compound C, the AMPK inhibitor did not reverse the anti-fibrotic effect of metformin on cell activation and collagen synthesis. And AICAR, another AMPK activator, did not suppress the cell activation and collagen synthesis stimulated by TGF-β1. These findings suggested that metformin might alleviate intestinal fibrosis via AMPK-independent pathways. Recently, mounting reports had concentrated on the AMPK-independent effects of metformin. Some studies (Xiao et al., 2010; Daskalopoulos et al., 2016) have indicated that metformin attenuated cardiac fibrosis independent of AMPK activation. And it was also indicated that metformin inhibited renal fibrosis via both AMPK-independent and dependent manners (Feng et al., 2017). The variability probably reflects the potent tissue- or cell-specific effects of metformin. Meanwhile, we identified only one limited cell type, and cannot accurately represent the complexity of the anti-fibrotic effect of metformin *in vivo*.

Intestinal fibrosis is the result of pleiotropic action of many cell types. We only identified the effect of metformin on fibroblast in our study, but the effect of metformin on the activation of immune cells in the development of fibrosis was not verified and needed further exploration. Meanwhile, we focused on the classic TGF-β/Smad signaling. However, the effects of metformin on other profibrotic cytokines such as TNF-α and IL-13, and other pathways such as WNT signaling and more recently YAP/TAZ pathway (Piersma et al., 2015) also needed to be further evaluated. And all results in our study were validated at the protein level, mRNA level might offer further insight and need further verification.

## Conclusion

**FIGURE 8:**
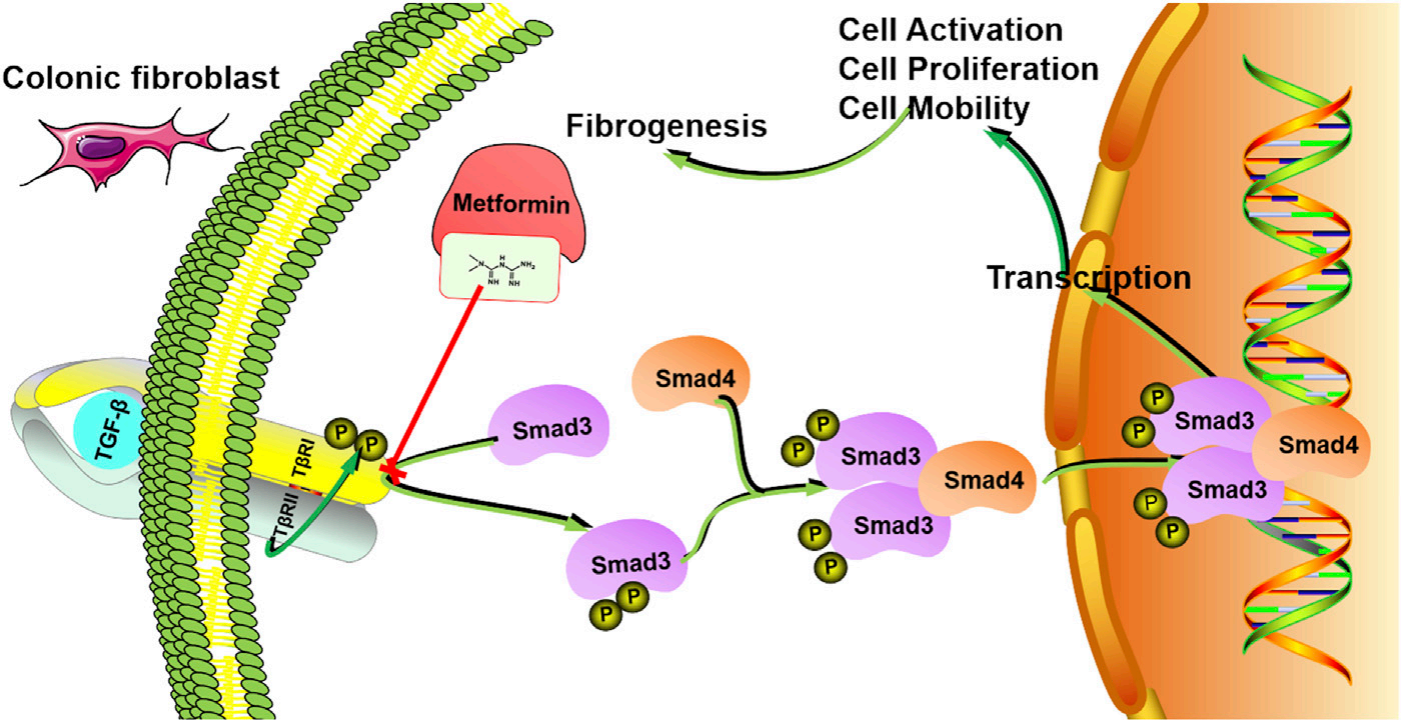
The mechanism model involved in the inhibitory effects of metformin in human intestinal fibrosis. Metformin suppresses the interaction between TβRI and Smad3 signaling in fibroblasts, leading to inhibition of cell activation, proliferation and mobility, finally inducing anti-fibrotic effects.

Taken together, we demonstrated that metformin protected against intestinal fibrosis induced by TNBS or DSS *in vivo* and inhibited activation and collagen synthesis in colon fibroblasts. The underlying mechanisms may be associated with the inhibition of interaction between TβRI and Smad3 (Figure 8). These findings provide the evidence of the application of metformin as a new therapeutic medicine for intestinal fibrosis.

## Data Availability

The original contributions presented in the study are included in the article/Supplementary Material, further inquiries can be directed to the corresponding author.

## Abbreviations

AICAR, 5-Aminoimidazole-4-carboxamide riboside; AMPK, AMP-activated protein kinas; CD, Crohn’s disease; DSS, Dextran sulfate sodium salt; ECM, Extracellular matrix; IBD, Inflammatory bowel disease; Smad, Small mothers against decapentaplegic; TGF-β1, Transforming growth factor-beta 1; TNBS, 2,4,6-Trinitro benzene sulfonic acid; TβRI, Transforming growth factor-beta receptor I; UC, Ulcerative colitis.

## References

[B1] Asensio-Lopez M. D. C. Lax A. Fernandez Del Palacio M. J. Sassi Y. Hajjar R. J. Pascual-Figal D. A. (2018). Pharmacological Inhibition of the Mitochondrial NADPH Oxidase 4/PKCα/Gal-3 Pathway Reduces Left Ventricular Fibrosis Following Myocardial Infarction. Transl Res. 199, 4–23. 10.1016/j.trsl.2018.04.004 29753686

[B2] Bai J. Zhang N. Hua Y. Wang B. Ling L. Ferro A. (2013). Metformin Inhibits Angiotensin II-Induced Differentiation of Cardiac Fibroblasts into Myofibroblasts. Plos One 8, e72120. 10.1371/journal.pone.0072120 24023727 PMC3759374

[B3] Bessissow T. Reinglas J. Aruljothy A. Lakatos P. L. Van Assche G. (2018). Endoscopic Management of Crohn's Strictures. World J. Gastroenterol. 24, 1859–1867. 10.3748/wjg.v24.i17.1859 29740201 PMC5937203

[B4] Bettenworth D. Bokemeyer A. Baker M. Mao R. Parker C. E. Nguyen T. (2019). Assessment of Crohn's Disease-Associated Small Bowel Strictures and Fibrosis on Cross-Sectional Imaging: A Systematic Review. Gut 68, 1115–1126. 10.1136/gutjnl-2018-318081 30944110 PMC6580870

[B5] Bouhnik Y. Carbonnel F. Laharie D. Stefanescu C. Hébuterne X. Abitbol V. (2018). Efficacy of Adalimumab in Patients with Crohn's Disease and Symptomatic Small Bowel Stricture: a Multicentre, Prospective, Observational Cohort (CREOLE) Study. Gut 67, 53–60. 10.1136/gutjnl-2016-312581 28119352 PMC5754855

[B6] Chan W. P. W. Mourad F. Leong R. W. (2018). Crohn's Disease Associated Strictures. J. Gastroenterol. Hepatol. 33, 998–1008. 10.1111/jgh.14119 29427364

[B7] Cheng D. Xu Q. Wang Y. Li G. Sun W. Ma D. (2021). Metformin Attenuates Silica-Induced Pulmonary Fibrosis via AMPK Signaling. J. Transl. Med. 19, 349. 10.1186/s12967-021-03036-5 34399790 PMC8365894

[B8] Daskalopoulos E. P. Dufeys C. Bertrand L. Beauloye C. Horman S. (2016). AMPK in Cardiac Fibrosis and Repair: Actions beyond Metabolic Regulation. J. Mol. Cel Cardiol. 91, 188–200. 10.1016/j.yjmcc.2016.01.001 26772531

[B9] Deng J. Zeng L. Lai X. Li J. Liu L. Lin Q. (2018). Metformin Protects against Intestinal Barrier Dysfunction via AMPKα1-dependent Inhibition of JNK Signalling Activation. J. Cel Mol. Med. 22, 546–557. 10.1111/jcmm.13342 PMC5742676 29148173

[B10] Derynck R. Zhang Y. E. (2003). Smad-dependent and Smad-independent Pathways in TGF-Beta Family Signalling. Nature 425, 577–584. 10.1038/nature02006 14534577

[B11] Fan K. Wu K. Lin L. Ge P. Dai J. He X. (2017). Metformin Mitigates Carbon Tetrachloride-Induced TGF-β1/Smad3 Signaling and Liver Fibrosis in Mice. Biomed. Pharmacother. 90, 421–426. 10.1016/j.biopha.2017.03.079 28390311

[B12] Feng Y. Wang S. Zhang Y. Xiao H. (2017). Metformin Attenuates Renal Fibrosis in Both AMPKα2-dependent and Independent Manners. Clin. Exp. Pharmacol. Physiol. 44, 648–655. 10.1111/1440-1681.12748 28273365

[B13] Ferguson M. W. Duncan J. Bond J. Bush J. Durani P. So K. (2009). Prophylactic Administration of Avotermin for Improvement of Skin Scarring: Three Double-Blind, Placebo-Controlled, Phase I/II Studies. Lancet 373, 1264–1274. 10.1016/S0140-6736(09)60322-6 19362676

[B14] Fiocchi C. Lund P. K. (2011). Themes in Fibrosis and Gastrointestinal Inflammation. Am. J. Physiol. Gastrointest. Liver Physiol. 300, G677–G683. 10.1152/ajpgi.00104.2011 21415411 PMC3094134

[B15] Hansel S. L. McCurdy J. D. Barlow J. M. Fidler J. Fletcher J. G. Becker B. (2018). Clinical Benefit of Capsule Endoscopy in Crohn's Disease: Impact on Patient Management and Prevalence of Proximal Small Bowel Involvement. Inflamm. Bowel Dis. 24, 1582–1588. 10.1093/ibd/izy050 29788055

[B16] Johnson P. Beswick E. J. Chao C. Powell D. W. Hellmich M. R. Pinchuk I. V. (2016). Isolation of CD 90+ Fibroblast/Myofibroblasts from Human Frozen Gastrointestinal Specimens. J. Vis. Exp. 107, e53691. 10.3791/53691 PMC4781712 26863470

[B17] Kheirollahi V. Wasnick R. M. Biasin V. Vazquez-Armendariz A. I. Chu X. Moiseenko A. (2019). Metformin Induces Lipogenic Differentiation in Myofibroblasts to Reverse Lung Fibrosis. Nat. Commun. 10, 2987. 10.1038/s41467-019-10839-0 31278260 PMC6611870

[B18] Latella G. Rogler G. Bamias G. Breynaert C. Florholmen J. Pellino G. (2014). Results of the 4th Scientific Workshop of the ECCO (I): Pathophysiology of Intestinal Fibrosis in IBD. J. Crohns Colitis 8, 1147–1165. 10.1016/j.crohns.2014.03.008 24731838

[B19] Lawrance I. C. Rogler G. Bamias G. Breynaert C. Florholmen J. Pellino G. (2017). Cellular and Molecular Mediators of Intestinal Fibrosis. J. Crohns Colitis 11, 1491–1503. 10.1016/j.crohns.2014.09.008 25306501 PMC5885809

[B20] Lee M. Katerelos M. Gleich K. Galic S. Kemp B. E. Mount P. F. (2018). Phosphorylation of Acetyl-CoA Carboxylase by AMPK Reduces Renal Fibrosis and Is Essential for the Anti-fibrotic Effect of Metformin. J. Am. Soc. Nephrol. 29, 2326–2336. 10.1681/ASN.2018010050 29976587 PMC6115654

[B21] Li G. Ren J. Hu Q. Deng Y. Chen G. Guo K. (2016). Oral Pirfenidone Protects against Fibrosis by Inhibiting Fibroblast Proliferation and TGF-β Signaling in a Murine Colitis Model. Biochem. Pharmacol. 117, 57–67. 10.1016/j.bcp.2016.08.002 27498142

[B22] Li K. Zhang T. T. Wang F. Cui B. Zhao C. X. Yu J. J. (2018). Metformin Suppresses Melanoma Progression by Inhibiting KAT5-Mediated SMAD3 Acetylation, Transcriptional Activity and TRIB3 Expression. Oncogene 37, 2967–2981. 10.1038/s41388-018-0172-9 29520103

[B23] Li J. Mao R. Kurada S. Wang J. Lin S. Chandra J. (2019). Pathogenesis of Fibrostenosing Crohn's Disease. Transl. Res. 209, 39–54. 10.1016/j.trsl.2019.03.005 30981697

[B24] Lin S. N. Mao R. Qian C. Bettenworth D. Wang J. Li J. (2021). Development of Anti-fibrotic Therapy in Stricturing Crohn's Disease: Lessons from Randomized Trials in Other Fibrotic Diseases. Physiol. Rev. 102, 605. 10.1152/physrev.00005.2021 34569264 PMC8742742

[B25] Macias M. J. Martin-Malpartida P. Massagué J. (2015). Structural Determinants of Smad Function in TGF-β Signaling. Trends Biochem. Sci. 40, 296–308. 10.1016/j.tibs.2015.03.012 25935112 PMC4485443

[B26] Maloy K. J. Powrie F. (2011). Intestinal Homeostasis and its Breakdown in Inflammatory Bowel Disease. Nature 474, 298–306. 10.1038/nature10208 21677746

[B27] Massagué J. (2012). TGFβ Signalling in Context. Nat. Rev. Mol. Cel Biol. 13, 616–630. 10.1038/nrm3434 PMC4027049 22992590

[B28] Noble P. W. Albera C. Bradford W. Z. Costabel U. Glassberg M. K. Kardatzke D. (2011). Pirfenidone in Patients with Idiopathic Pulmonary Fibrosis (CAPACITY): Two Randomised Trials. Lancet 377, 1760–1769. 10.1016/S0140-6736(11)60405-4 21571362

[B29] Piersma B. Bank R. A. Boersema M. (2015). Signaling in Fibrosis: TGF-β, WNT, and YAP/TAZ Converge. Front. Med. (Lausanne) 2, 59. 10.3389/fmed.2015.00059 26389119 PMC4558529

[B30] Rangarajan S. Bone N. B. Zmijewska A. A. Jiang S. Park D. W. Bernard K. (2018). Metformin Reverses Established Lung Fibrosis in a Bleomycin Model. Nat. Med. 24, 1121–1127. 10.1038/s41591-018-0087-6 29967351 PMC6081262

[B31] Rena G. Hardie D. G. Pearson E. R. (2017). The Mechanisms of Action of Metformin. Diabetologia 60, 1577–1585. 10.1007/s00125-017-4342-z 28776086 PMC5552828

[B32] Rieder F. Fiocchi C. Rogler G. (2017). Mechanisms, Management, and Treatment of Fibrosis in Patients with Inflammatory Bowel Diseases. Gastroenterology 152, 340–e6. 10.1053/j.gastro.2016.09.047 27720839 PMC5209279

[B33] Sato N. Takasaka N. Yoshida M. Tsubouchi K. Minagawa S. Araya J. (2016). Metformin Attenuates Lung Fibrosis Development via NOX4 Suppression. Respir. Res. 17, 107. 10.1186/s12931-016-0420-x 27576730 PMC5006432

[B34] Scheibe K. Kersten C. Schmied A. Vieth M. Primbs T. Carlé B. (2019). Inhibiting Interleukin 36 Receptor Signaling Reduces Fibrosis in Mice with Chronic Intestinal Inflammation. Gastroenterology 156, 1082. 10.1053/j.gastro.2018.11.029 30452921

[B35] Trachtman H. Fervenza F. C. Gipson D. S. Heering P. Jayne D. R. Peters H. (2011). A Phase 1, Single-Dose Study of Fresolimumab, an Anti-TGF-β Antibody, in Treatment-Resistant Primary Focal Segmental Glomerulosclerosis. Kidney Int. 79, 1236–1243. 10.1038/ki.2011.33 21368745 PMC3257033

[B36] Vernier-Massouille G. Balde M. Salleron J. Turck D. Dupas J. L. Mouterde O. (2008). Natural History of Pediatric Crohn's Disease: A Population-Based Cohort Study. Gastroenterology 135, 1106–1113. 10.1053/j.gastro.2008.06.079 18692056

[B37] Wang M. Chen D. Q. Chen L. Cao G. Zhao H. Liu D. (2018). Novel Inhibitors of the Cellular Renin-Angiotensin System Components, Poricoic Acids, Target Smad3 Phosphorylation and Wnt/β-Catenin Pathway against Renal Fibrosis. Br. J. Pharmacol. 175, 2689–2708. 10.1111/bph.14333 29679507 PMC6003649

[B38] Wang J. Lin S. Brown J. M. van Wagoner D. Fiocchi C. Rieder F. (2021). Novel Mechanisms and Clinical Trial Endpoints in Intestinal Fibrosis. Immunol. Rev. 302, 211–227. 10.1111/imr.12974 33993489 PMC8292184

[B39] Wilcock C. Bailey C. J. (1994). Accumulation of Metformin by Tissues of the normal and Diabetic Mouse. Xenobiotica 24, 49–57. 10.3109/00498259409043220 8165821

[B40] Wirtz S. Popp V. Kindermann M. Gerlach K. Weigmann B. Fichtner-Feigl S. (2017). Chemically Induced Mouse Models of Acute and Chronic Intestinal Inflammation. Nat. Protoc. 12, 1295–1309. 10.1038/nprot.2017.044 28569761

[B41] Xiao H. Ma X. Feng W. Fu Y. Lu Z. Xu M. (2010). Metformin Attenuates Cardiac Fibrosis by Inhibiting the TGFbeta1-Smad3 Signalling Pathway. Cardiovasc. Res. 87, 504–513. 10.1093/cvr/cvq066 20200042

[B42] Yan J. B. Lai C. C. Jhu J. W. Gongol B. Marin T. L. Lin S. C. (2020). Insulin and Metformin Control Cell Proliferation by Regulating TDG-Mediated DNA Demethylation in Liver and Breast Cancer Cells. Mol. Ther. Oncolytics 18, 282–294. 10.1016/j.omto.2020.06.010 32728616 PMC7378318

[B43] Zhou D. M. Ran F. Ni H. Z. Sun L. L. Xiao L. Li X. Q. (2020). Metformin Inhibits High Glucose-Induced Smooth Muscle Cell Proliferation and Migration. Aging (Albany NY) 12, 5352–5361. 10.18632/aging.102955 32208365 PMC7138554

