# Supplementary material for: Metformin Ameliorates Chronic Colitis-Related Intestinal Fibrosis via Inhibiting TGF-β1/Smad3 Signaling
Source: Front Pharmacol. 2022 May 13;13:887497. doi: 10.3389/fphar.2022.887497 (PMC9136141; doi:10.3389/fphar.2022.887497)
Supplement: Supplementary file 1 [file Table1.DOCX]

Download links of full original source data：

<https://www.jianguoyun.com/p/DTq8uV8Q8PT9CRjJkrAE>
