# Supplementary material for: Metformin Ameliorates Chronic Colitis-Related Intestinal Fibrosis via Inhibiting TGF-β1/Smad3 Signaling
Source: Front Pharmacol. 2022 May 13;13:887497. doi: 10.3389/fphar.2022.887497 (PMC9136141; doi:10.3389/fphar.2022.887497)
Supplement: Supplementary file 2 [file DataSheet1.ZIP › Supplementary materials/Supplementary figure legend.docx]

**Fig. S1 Control staining using PBS in immunohistochemical analyses.** (**A-B**) PBS-staining figures of preventive metformin application in TNBS (**A**) and DSS (**B**) mice model. (**C-D**) PBS-staining figures of therapeutic metformin application in TNBS (**C**) and DSS(**D**) mice model. Scale bars, 100 μm.

**Fig. S2 Annexin V/PI staining allows separation of early apoptotic, non-viable and healthy cell populations.** CCD-18Co cells were (**A**) untreated, (**B**) stimulated with rhTGF-β1 (2 ng/mL), (**C-D**) stimulated with rhTGF-β1 (2 ng/mL) after pre-incubation with 0.1 mM (**C**) or 0.5 mM (**D**) metformin for 4 h. The figures exhibit one representative data set from three independent experiments. (**E**) The data show mean percent of apoptotic cell of three independent experiments described in (**A-D**).

**Fig. S3 Fibrosis and collagen deposition are increased in mucosa and submucosa layers of stenotic tissues of CD patients and TGF-β1/Smad3 pathways are activated.** (**A**) Representative H&E-stained pictures of stenotic tissues and control specimens were indicated. Bars show the minimal and maximal thicknesses of the submucosa layer. The absolute thickness of the submucosa was quantified in relative normal tissues (n = 18) and stenotic ones (n = 18). (**B**) The relative min and max thickness of the mucosa, the inner and outer layers of muscularis propria, were also quantified in H&E-stained colon sections as above. (**C**) Hydroxyproline content from intestine surgical samples of relative normal tissues (n = 18) and stenotic ones (n = 18) was analyzed. (**D**) Representative Sirius Red-stained images (n = 3 independent experiments) are shown. (**E-F**) Fibrotic markers and proteins in TGF-β1/Smad3 pathways were evaluated by western blot and immunohistochemical analyses in intestinal tissues as indicated (n = 3 samples /group). Representative IHC images are shown. (**G**) Representative images of IF staining conducted with antibodies against Vimentin and α-SMA, markers of myofibroblasts. White arrowheads show the co-expression of Vimentin and α-SMA. Difference analysis between relative normal tissues and stenotic groups were performed with unpaired Student’s t test (∗, P <0.05; ∗∗, P < 0.005; ∗∗∗, P <0.0005; 2-tailed.). Means ± SD. Scale bars, (**A**) 1 mm; (**D**) 300 µm; (**F** and **G**) 200 µm.
