# Supplementary figures and images for: Metformin Ameliorates Chronic Colitis-Related Intestinal Fibrosis via Inhibiting TGF-β1/Smad3 Signaling
Source: Front Pharmacol. 2022 May 13;13:887497. doi: 10.3389/fphar.2022.887497 (PMC9136141; doi:10.3389/fphar.2022.887497)

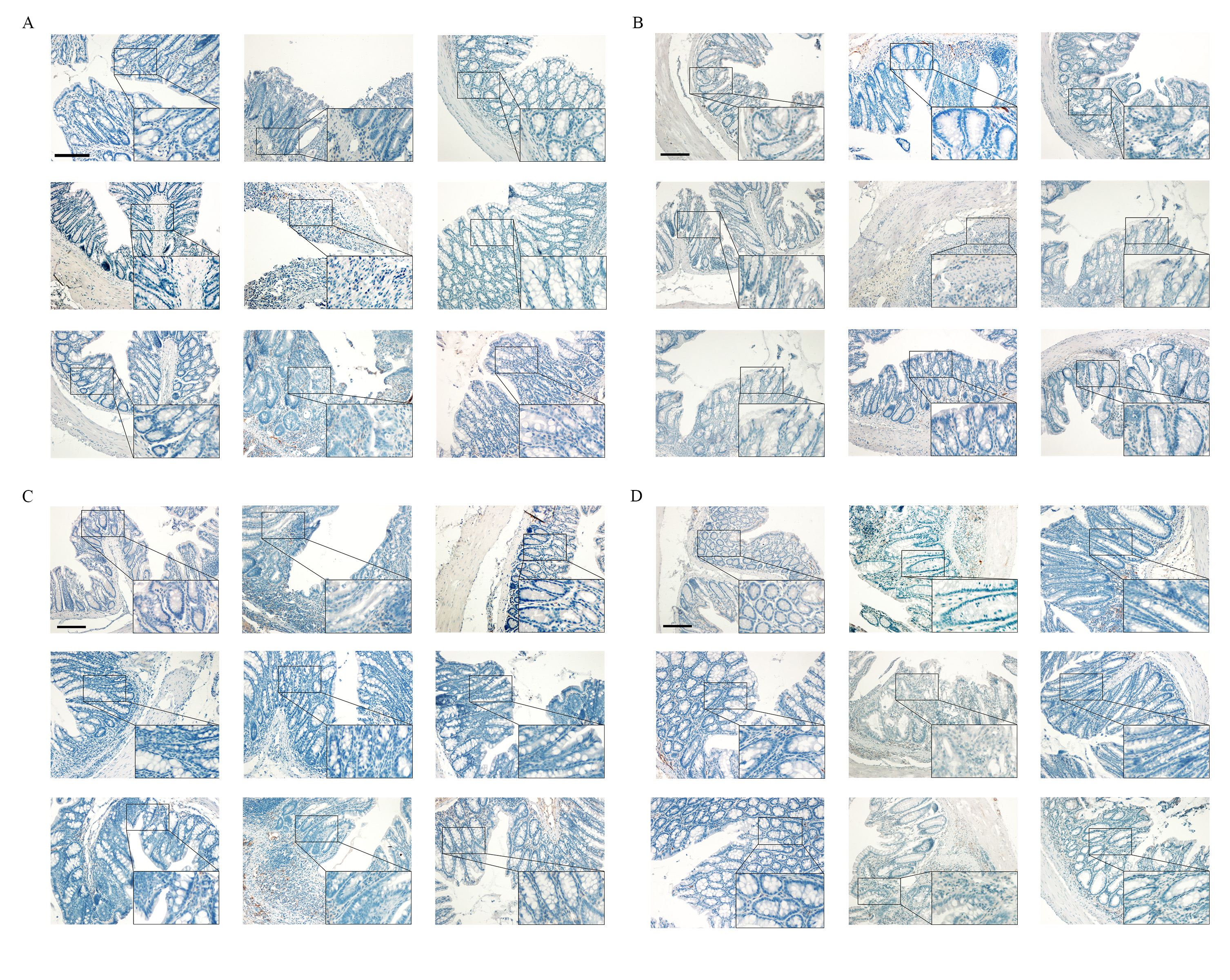

Supplement: Supplementary file 2 [file DataSheet1.ZIP › Supplementary materials/Supplementary Figure S1.jpg]

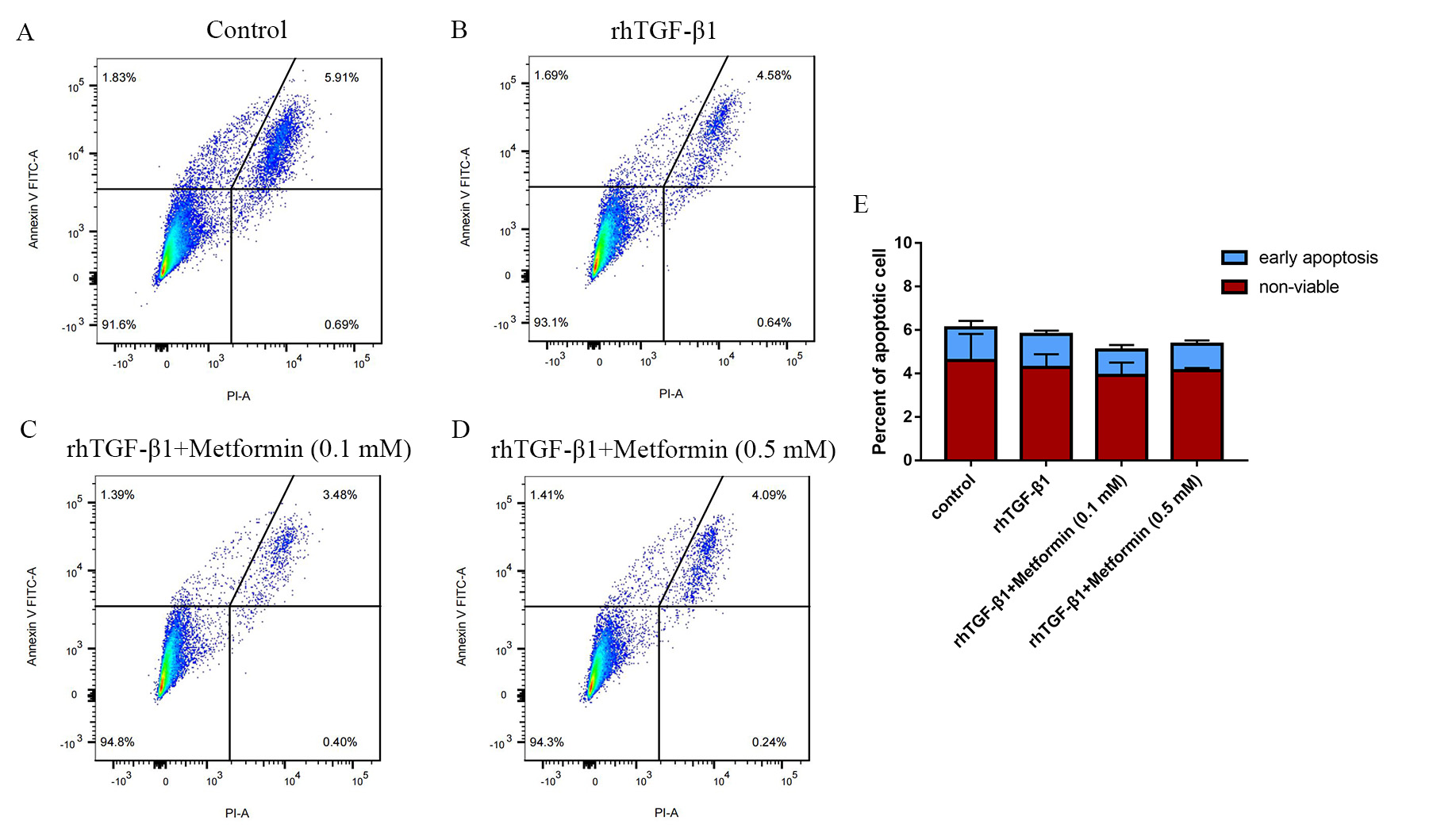

Supplement: Supplementary file 2 [file DataSheet1.ZIP › Supplementary materials/Supplementary Figure S2.jpg]

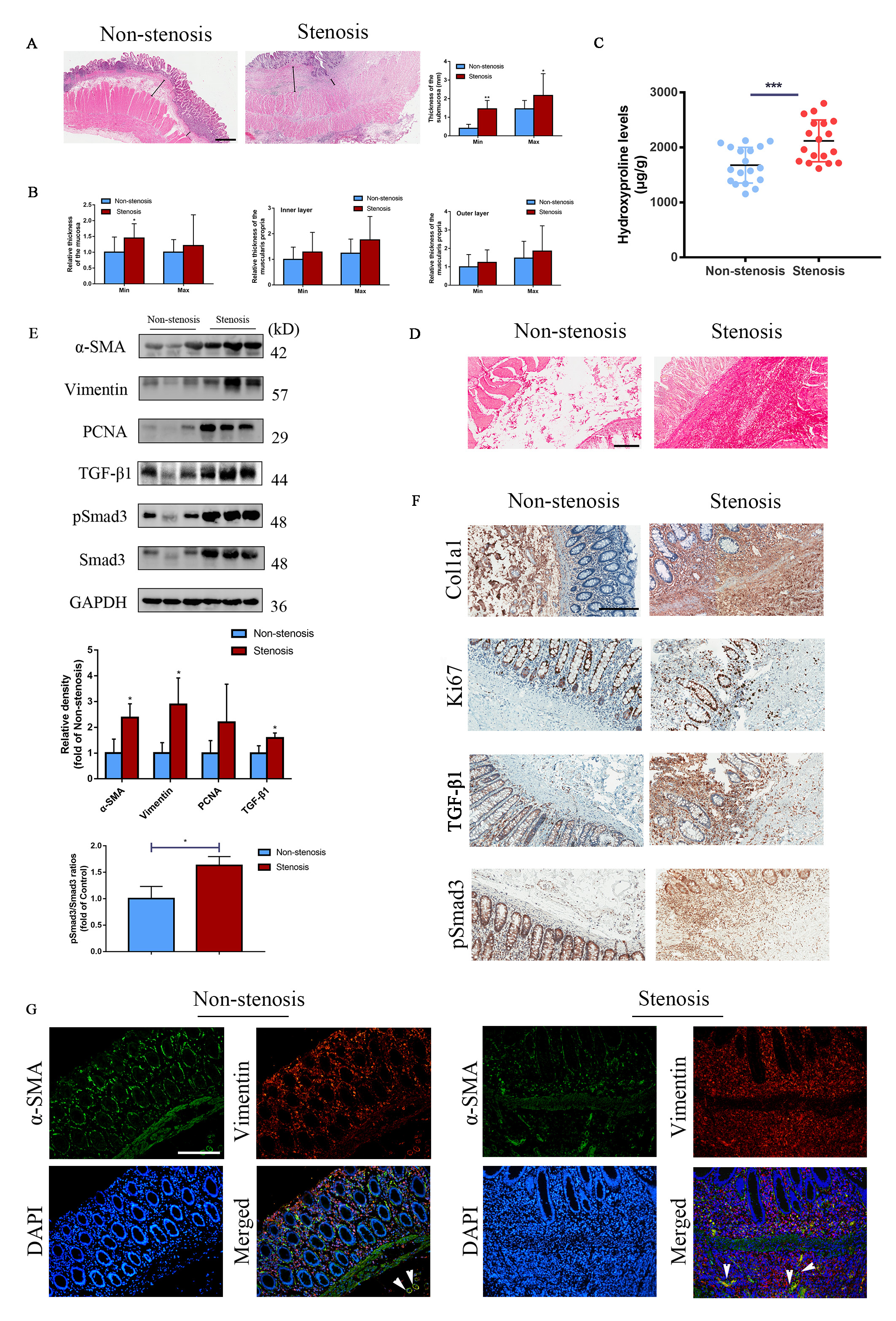

Supplement: Supplementary file 2 [file DataSheet1.ZIP › Supplementary materials/Supplementary Figure S3.jpg]
